# A case report on the usefulness of combining online continuous wavelet transform analysis with a novel real-time phase mapping system during nonparoxysmal atrial fibrillation catheter ablation

**DOI:** 10.1016/j.hrcr.2022.01.004

**Published:** 2022-01-19

**Authors:** Yasuhiro Yokoyama, Hiroaki Nakamura, Nobutaka Kato, Koji Higuchi

**Affiliations:** ∗ Department of Cardiology, St Luke’s International Hospital, Tokyo, Japan; † Department of Cardiology, Hiratsuka Kyosai Hospital, Hiratsuka, Japan

**Keywords:** Atrial fibrillation, Catheter ablation, Atrial fibrillation driver, Continuous wavelet transform analysis, Phase mapping

## Introduction

Studies are still ongoing to find a perpetuator for atrial fibrillation (AF), the AF driver. The continuous wavelet transform (CWT) analysis is a frequency analysis that can evaluate the temporal stability of the derived frequency (pseudo-frequency).^1^ We have previously reported the utility of temporally stable pseudo-frequency (sPF) derived from off-line CWT analysis for nonparoxysmal AF catheter ablation.^1^^,^^2^ A novel real-time phase mapping system (ExTRa Mapping; Nihon Kohden, Tokyo, Japan) that displays local AF wave dynamics has been developed, and its use is spreading in Japan.^3^, ^4^, ^5^, ^6^ Recently, an online CWT analysis combined with ExTRa Mapping during catheter ablation became available. This is the first report to demonstrate that sPF can guide the limited field of view of the ExTRa Mapping to detect an AF driver in the human heart.

## Case report

A 56-year-old Japanese man with persistent AF who had a coronary artery bypass graft and was on regular hemodialysis underwent initial pulmonary vein isolation (PVI) using cryoballoon catheter ablation under general anesthesia. The patient did not receive any antiarrhythmic drugs. Informed consent was obtained before catheter ablation. This case was presented in accordance with the Declaration of Helsinki. No linear ablation was added except for the cavotricuspid isthmus block line after restoring sinus rhythm.

Before catheter ablation, a bipolar electrogram was recorded during AF for 30 seconds using a 20-pole spiral-shaped mapping catheter of 2.5 cm diameter (Reflexion HD; Abbott, Chicago, IL) at selected sites in both atria (right atrium: superior vena cava, appendage, crista terminalis, cavotricuspid isthmus, coronary sinus ostium, septum, and sinus venosus; left atrium: pulmonary veins, roof, posterior, inferior, mitral isthmus, floor, appendage, anterior, septum). The images of the mapping catheter at each recording site were stored on a 3-dimensional (3D) electroanatomical mapping system (EnSite NavX System; Abbott, Chicago, IL) to display later. The recorded bipolar electrogram data were stored on a polygraph (Cath Lab RMC 5000; Nihon Koden, Tokyo, Japan). Then, an online CWT analysis from the entire 30 seconds of the bipolar electrogram was performed at each site, and simultaneously, real-time phase mapping using ExTRa Mapping from the last 5 seconds of the same bipolar electrogram was performed.

**Figure 1:**
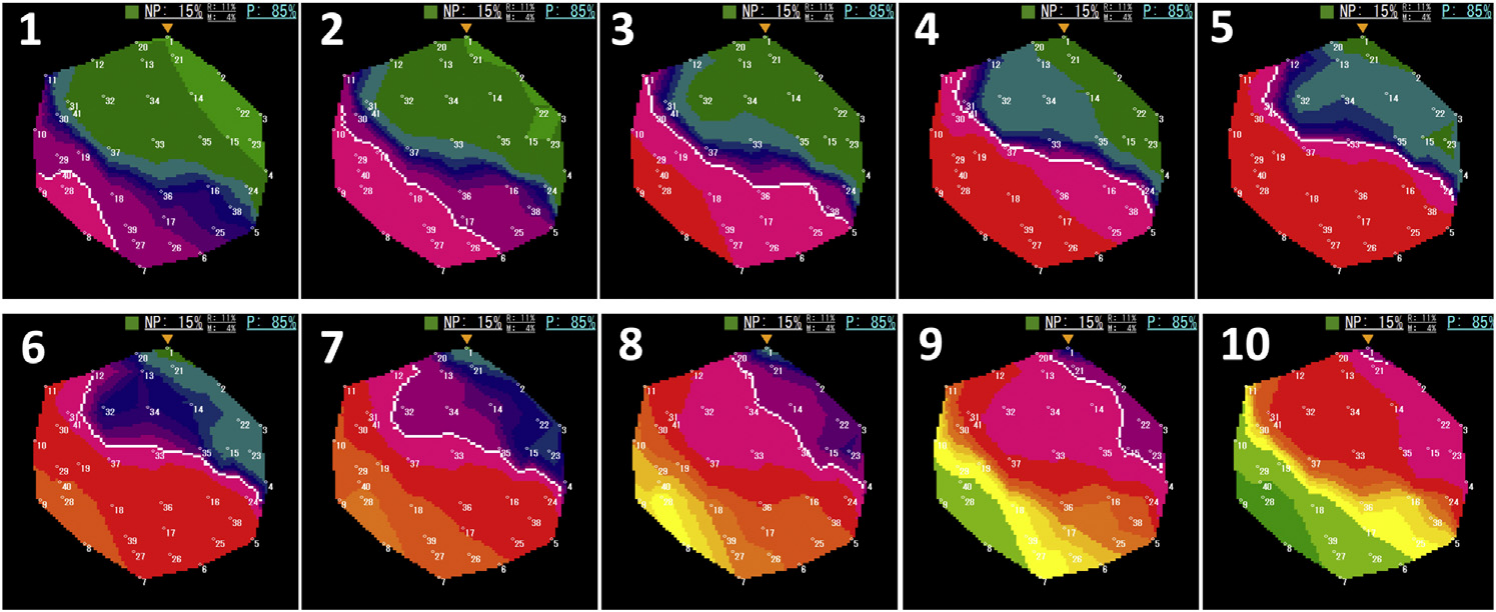
Frame advance of representative atrial fibrillation wave dynamics at left inferior pulmonary vein antrum. Of the 5-second phase map movie, the activation sequence of the representative episodes during 400 ms (40 ms × 10 consecutive time windows) is depicted in frame advance. The numbers on each movie screen indicate 41 bipolar electrograms (1–32 are physical bipolar, 33–41 are virtual bipolar). The green square at the top of each screen shows the reliability of ExTRa Mapping (Nihon Kohden, Tokyo, Japan). The color is determined according to the number of rejected bipolar electrograms owing to their low quality: blue, 0; green, 1; yellow, 2–4; orange, 5–10; red, 11–16; and gray, ≥17. The colors change in real time on the screen, informing the operator of the reliability of ExTRa Mapping. The nonpassively activated ratio was only adopted when the reliability was high enough, such as blue, green, and yellow. White lines indicate the head of the wavefront. The wavefront traveled from the northwest to the southeast (from 1 to 10) on the screen. This activation sequence appeared intermittently and repeatedly during the phase map movie. M = multiple wavelets; NP = nonpassive activation; P = passive activation; R = rotors.

**Figure 2:**
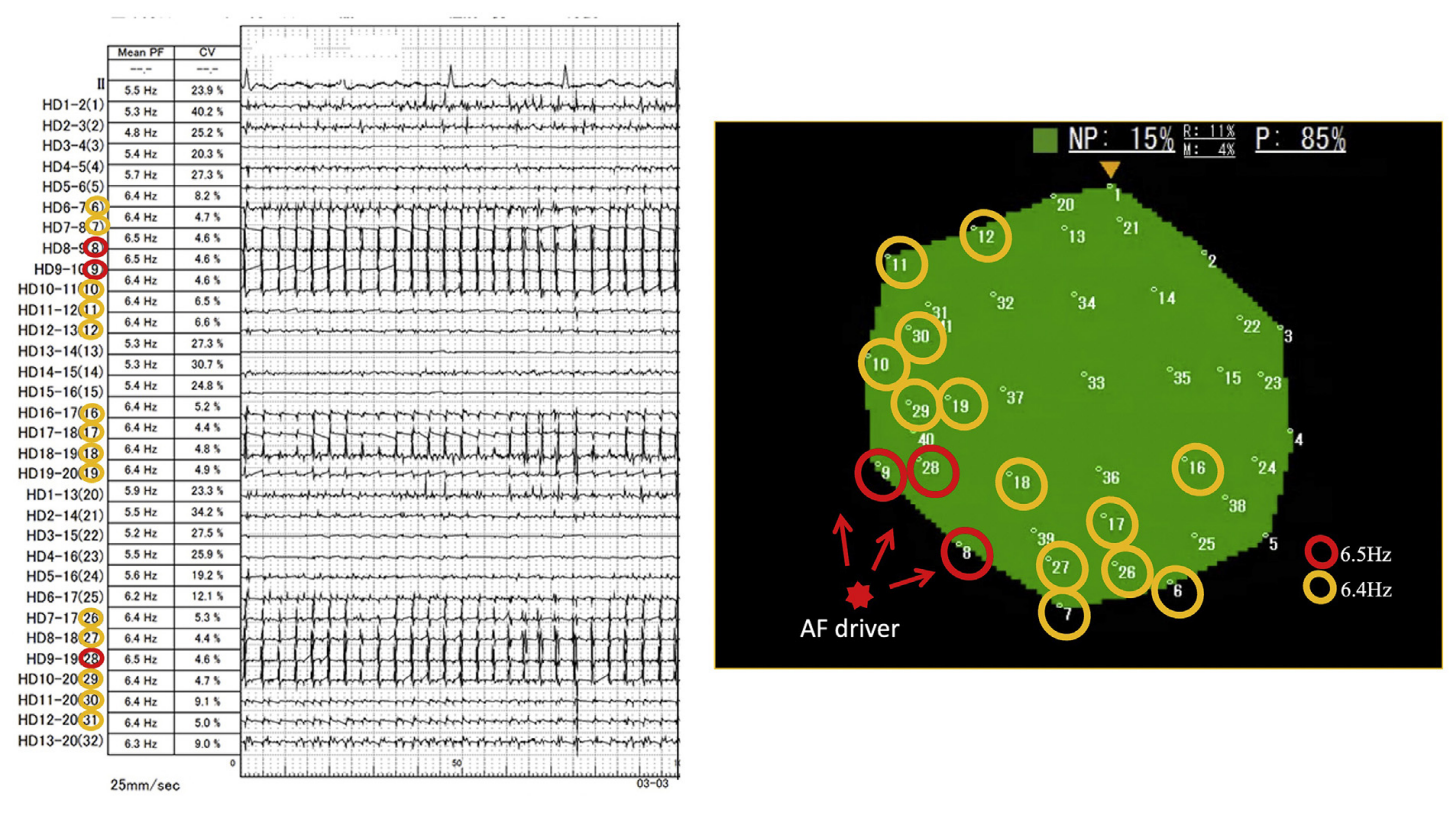
Continuous wavelet transform analysis results and phase map movie screen at left inferior pulmonary vein antrum during atrial fibrillation (AF) before pulmonary vein isolation. Left panel: Recordings from the top are electrocardiography lead II and physical bipolar electrograms recorded from the 20-pole spiral-shaped catheter (HD1-2 to HD13-20). The numbers in parentheses are the numbers 1–32, assigned to the physical bipolar electrograms. The second column shows the pseudo-frequency (PF), and the third column shows the coefficient of variation (CV). The sweep speed was 25 mm/s. Right panel: Phase map movie screen where the indications of the numbers on the screen are the same as those in Figure 1. The meaning of the green-colored square at the top of the phase map movie screen is the same as in Figure 1. The red star on the northwest side of the screen was the estimated AF driver location. The red arrow indicates the direction of the wave from the AF driver to the phase map movie screen. In both panels, the red open circle is the highest stable PF (sPF = PF with CV <10)^1^ at 6.5 Hz, and the orange open circle is the second-highest sPF at 6.4 Hz. Other abbreviations as in Figure 1.

**Figure 3:**
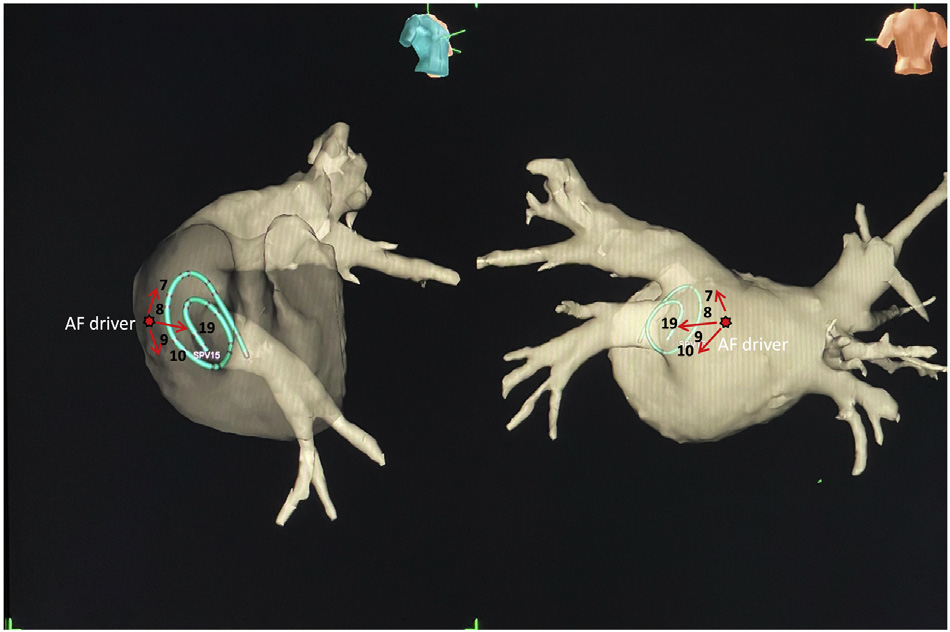
The placement of the 20-pole spiral-shaped mapping catheter at the left inferior pulmonary vein antrum in a 3-dimensional (3D) electroanatomical mapping image. The estimated location of the atrial fibrillation (AF) driver is shown in the 3D electroanatomical image of the patient. The number next to the 20-pole spiral-shaped catheter in the image indicates the electrode number. Red star indicates the estimated AF driver location. The red arrow indicates the direction of the AF wave from the AF driver. Adjusting the spiral 20-pole electrode catheter placement more posteriorly may have resulted in a higher nonpassively activated ratio on ExTRa Mapping (Nihon Kohden, Tokyo, Japan).

CWT analysis revealed the highest sPF (6.5 Hz), which is the main AF driver site, at the left inferior pulmonary vein (LIPV) antrum (Supplemental Table 1). The nonpassively activated ratio (%NP), which is the ratio of the rotors’ and multiple wavelets’ appearance time to the recording time, automatically calculated on the phase map movie of the ExTRa Mapping,^3^, ^4^, ^5^, ^6^ was expected to be high at this site but was only 15% (Figures 1 and 2). However, tagging the bipolar electrogram numbers showing sPF according to their frequencies on the phase map movie screen revealed a frequency gradient from northwest to southeast (Figure 2). Thus, considering the direction of the AF wave dynamics and frequency gradient, the main AF driver would exist on the northwest outside the phase map movie screen (Supplemental Movie 1). That is, placing the 20-pole spiral-shaped catheter more posteriorly from that site on the 3D electroanatomical mapping image could have revealed a higher %NP (Figure 3). Notably, the frequency gradient at this site was ambiguous when using the dominant frequency (DF) derived from the fast Fourier transform (FFT) analysis based on the same 5-second bipolar electrogram as for the phase mapping (Supplemental Figure 1).

Cardioversion was needed after PVI to restore sinus rhythm, although the LIPV antrum site with the highest sPF was included in the cryoballoon ablation area. The prolonged mean AF cycle length after PVI (161–166 ms and 169–190 ms at the left atrium lateral and right atrium lateral, respectively) indicates that an AF driver with a lower frequency might have sustained AF. After this catheter ablation session, the patient maintained sinus rhythm without any antiarrhythmic drugs for more than 3 months.

## Discussion

An AF driver is elegantly described in an experimental animal model.^7^^,^^8^ In the AF-induced Langendorff-perfused sheep heart, the distribution of DF derived from FFT analysis was examined. Then, the optical mapping identifies a periodic atrial activity lasting for a period of 20−30 minutes near the DF sites. Relatively high DF sites with periodic atrial activities would indicate AF drivers.^7^

Many clinical studies have used DF to detect AF drivers, usually derived from a 5-second recording of bipolar AF electrogram.^9^, ^10^, ^11^ However, the current expert consensus statement on AF ablation states that “DF-based ablation strategy is of unknown usefulness for AF ablation.”^12^ On the other hand, AF drivers have been reported temporally stable in certain areas.^7^^,^^13^ FFT analysis theoretically cannot guarantee the temporal stability of the derived DF. Thus, DF alone could be insufficient for use as a surrogate for an AF driver during catheter ablation. CWT analysis is another frequency analysis that can prove the temporal stability of the frequency during AF as an sPF. From a direct comparison of DF and sPF during nonparoxysmal AF catheter ablation, we reported that the highest sPF sites are more reliable than the highest DF sites for estimating the main AF driver sites.^1^ However, for more precise AF driver detection, periodic atrial activity patterns such as rotors and multiple wavelets must also be identified in addition to sPF.

ExTRa Mapping is a real-time phase mapping system that displays local AF wave dynamics. It is based on 41 bipolar intra-atrial electrograms (32 physical bipolar and 9 virtual bipolar electrograms) recorded for 5 seconds during AF using a 20-pole spiral-shaped mapping catheter with a 2.5-cm diameter as a mapping catheter.^3^, ^4^, ^5^, ^6^ The mapping accuracy of ExTRa Mapping against optical mapping has been proven in an experimental animal study.^5^ According to ExTRa Mapping before PVI in both atria in our patient, the highest % NP of 70% was detected at the crista terminalis (Supplemental Movie 2). However, sPF at the crista terminalis was 5.6 Hz, far lower than the highest sPF, 6.5 Hz, at the LIPV antrum (Supplemental Table 1). These findings reveal the pitfall of AF driver detection based solely on %NP data.

CWT analysis and ExTRa Mapping can complement each other and reproduce the conditions under which the AF driver was detected in the experimental animal model. That is, CWT analysis identifies high frequencies as sPF, and ExTRa Mapping displays specific periodic atrial activity that indicates AF drivers near the high-sPF sites. The only difference is that the temporal stability of the periodic atrial activity would be guaranteed via sPF, not visually, as in the experimental animal model. However, it must be noted that the field of view for ExTRa Mapping is limited to the size of the mapping catheter (2.5 cm in diameter) rather than the panoramic field of view, as in the previously reported clinical phase mapping systems.^13^, ^14^, ^15^ Therefore, it is crucial that the mapping catheter adequately cover the AF driver. If the AF driver is located at the edge of or outside the field of view of ExTRa Mapping, the %NP will be naturally low. Our case proffers a solution to this issue. Even at sites with low %NP, the presence of relatively high sPF and its gradient distribution along the AF wave dynamics would help estimate the location of the AF driver in the vicinity of the field of view of the ExTRa Mapping. The combination of online CWT analysis and ExTRa Mapping is expected to be a new method of AF driver detection.

## Conclusion

The combination of online CWT analysis and ExTRa Mapping would be useful for AF driver detection during nonparoxysmal AF catheter ablation. However, further clinical validation is required to assess the effectiveness of this combination.

## Appendix Supplementary data

Supplemental Movie 1 Movie of representative atrial fibrillation (AF) wave dynamics at left inferior pulmonary vein antrum before pulmonary vein isolation. The movie shows the AF dynamics shown in Figure 1. The indications of the numbers, white lines, open circles, red stars, and red arrows on the movie screen are the same as in Figures 1 and 2. The meaning of the green-colored square at the top of the phase map movie screen is the same as in Figure 1. The wavefront traveled intermittently and repeatedly from northwest to southeast. Abbreviations are the same as in Figures 1 and 2. (Note: This is a still image. The movie will be played on the attached PowerPoint file.)

Supplemental Movie 2 Continuous wavelet transfrom analysis results and phase map movie screen at crista terminals during atrial fibrillation before pulmonary vein isolation, at which the highest nonpassively activated ratio of 70% was detected in both atria. Left panel: Recordings from the top are electrocardiography lead II and physical bipolar electrograms recorded from the 20-pole spiral-shaped catheter (HD1-2 to HD13-20). The numbers in parentheses are the numbers 1 to 32 assigned to the physical bipolar electrograms. The second column shows the pseudo-frequency (PF), and the third column shows the coefficient of variation (CV). The sweep speed was 25 mm/s. Right panel: Phase map movie screen where the indications of the numbers on the screen are the same as those in Figure 1. The meaning of the green-colored square at the top of the phase map movie screen is the same as in Figure 1. The open circle represents stable PF (sPF = PF with CV <10)^1^ in both panels: red for 5.6 Hz, orange for 5.5 Hz, and yellow for 5.2 Hz. sPF at crista terminalis was far lower than the highest sPF of 6.5 Hz at left inferior pulmonary vein antrum. Abbreviations are the same as those in Figures 1 and 2. (Note: This is a still image. The movie will be played on the attached PowerPoint file.)

Supplemental Figure 1 Fast Fourier transform (FFT) analysis results and phase map movie screen at left inferior pulmonary vein antrum during atrial fibrillation (AF) before pulmonary vein isolation. Left panel: Recordings from the top are electrocardiography lead II and physical bipolar electrograms recorded from the 20-pole spiral-shaped catheter (HD1-2 to HD13-20). The numbers in parentheses are the same as those shown in Figure 2. The second column shows the dominant frequency (DF), and the third column shows the regularity index (RI). Only DFs with RI ≥0.2 were regarded as significant. The FFT analysis was performed using the same 5-s bipolar electrogram as for the phase mapping. The sweep speed was 25 mm/s. Right panel: Phase map movie screen where the indications of the numbers on the screen are the same as that in Figure 1. The meaning of the green-colored square at the top of the phase map movie screen is the same as in Figure 1. The open circle represents significant DFs in both panels, red for 6.8 Hz, orange for 6.6 Hz, yellow for 6.4 Hz, and blue for 6.2 Hz. Compared to Figure 2, the frequency gradient along the AF wave dynamics is ambiguous for estimating the location of the AF driver. Abbreviations are the same as those in Figures 1 and 2. **Supplemental Table 1****.** Sites of the stable pseudo-frequency (sPF) in both atria The sites where sPF [pseudo-frequency (PF) with coefficient of variation (CV) <10]^1^ was detected in both atria are shown in descending order. The meaning of the colors for the reliability of ExTRa Mapping (Nihon Kohden, Tokyo Japan) is the same as in Figure 1. %NP, nonpassively activated ratio; R, rotors; M, multiple wavelets; LAA, left atrial appendage; RIPV, right inferior pulmonary vein; LA, left atrium; LSPV, left superior pulmonary vein; RSPV, right superior pulmonary vein; CS, coronary sinus.
