# Supplementary material for: A case report on the usefulness of combining online continuous wavelet transform analysis with a novel real-time phase mapping system during nonparoxysmal atrial fibrillation catheter ablation
Source: HeartRhythm Case Rep. 2022 Jan 19;8(4):250–3. doi: 10.1016/j.hrcr.2022.01.004 (PMC9039114; doi:10.1016/j.hrcr.2022.01.004)
Supplement: Supplemental Figure 1 — Fast Fourier transform (FFT) analysis results and phase map movie screen at left inferior pulmonary vein antrum during atrial fibrillation (AF) before pulmonary vein isolation. Left panel: Recordings from the top are electrocardiography lead II and physical bipolar electrograms recorded from the 20-pole spiral-shaped catheter (HD1-2 to HD13-20). The numbers in parentheses are the same as those shown in Figure 2. The second column shows the dominant frequency (DF), and the third column shows the regularity index (RI). Only DFs with RI ≥0.2 were regarded as significant. The FFT analysis was performed using the same 5-s bipolar electrogram as for the phase mapping. The sweep speed was 25 mm/s. Right panel: Phase map movie screen where the indications of the numbers on the screen are the same as that in Figure 1. The meaning of the green-colored square at the top of the phase map movie screen is the same as in Figure 1. The open circle represents significant DFs in both panels, red for 6.8 Hz, orange for 6.6 Hz, yellow for 6.4 Hz, and blue for 6.2 Hz. Compared to Figure 2, the frequency gradient along the AF wave dynamics is ambiguous for estimating the location of the AF driver. Abbreviations are the same as those in Figures 1 and 2. Supplemental Table 1. Sites of the stable pseudo-frequency (sPF) in both atria The sites where sPF [pseudo-frequency (PF) with coefficient of variation (CV) <10]1 was detected in both atria are shown in descending order. The meaning of the colors for the reliability of ExTRa Mapping (Nihon Kohden, Tokyo Japan) is the same as in Figure 1. %NP, nonpassively activated ratio; R, rotors; M, multiple wavelets; LAA, left atrial appendage; RIPV, right inferior pulmonary vein; LA, left atrium; LSPV, left superior pulmonary vein; RSPV, right superior pulmonary vein; CS, coronary sinus. [file mmc3.docx]

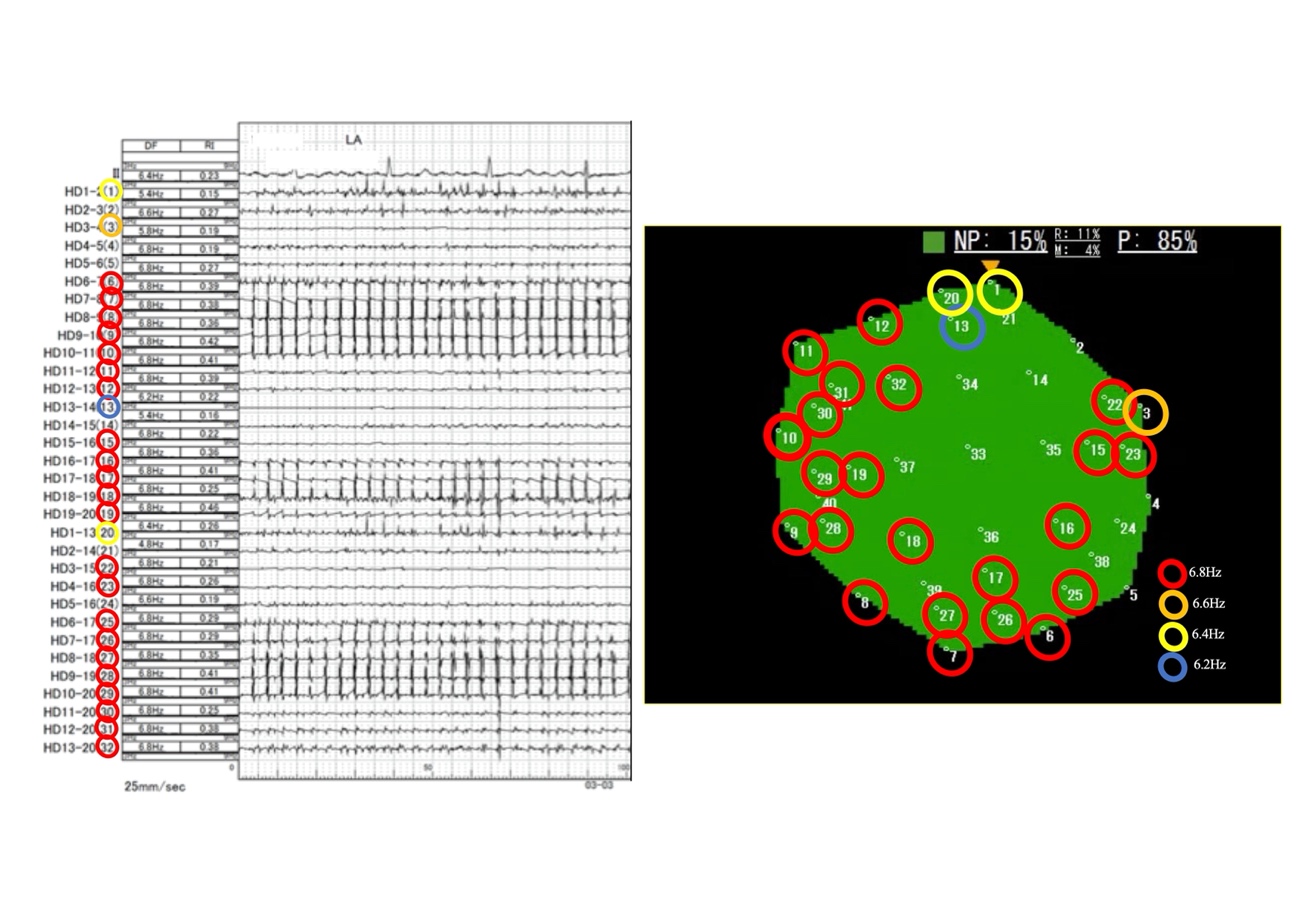


**Supplemental Figure.**

Fast Fourier transform (FFT) analysis results and phase map movie screen at left inferior pulmonary vein antrum during atrial fibrillation (AF) before pulmonary vein isolation.

Left panel: Recordings from the top are electrocardiography lead II and physical bipolar electrograms recorded from the 20-pole spiral-shaped catheter (HD1-2 to HD13-20). The numbers in parentheses are the same as those shown in Figure 2. The second column shows the dominant frequency (DF), and the third column shows the regularity index (RI). Only DFs with RI ≥0.2 were regarded as significant. The FFT analysis was performed using the same 5-s bipolar electrogram as for the phase mapping. The sweep speed was 25 mm/s. Right panel: Phase map movie screen where the indications of the numbers on the screen are the same as that in Figure 1. The meaning of the green-colored square at the top of the phase map movie screen is the same as in Figure 1. The open circle represents significant DFs in both panels, red for 6.8 Hz, orange for 6.6 Hz, yellow for 6.4 Hz, and blue for 6.2 Hz. Compared to Figure 2, the frequency gradient along the AF wave dynamics is ambiguous for estimating the location of the AF driver.

Abbreviations are the same as those in Figures 1 and 2.


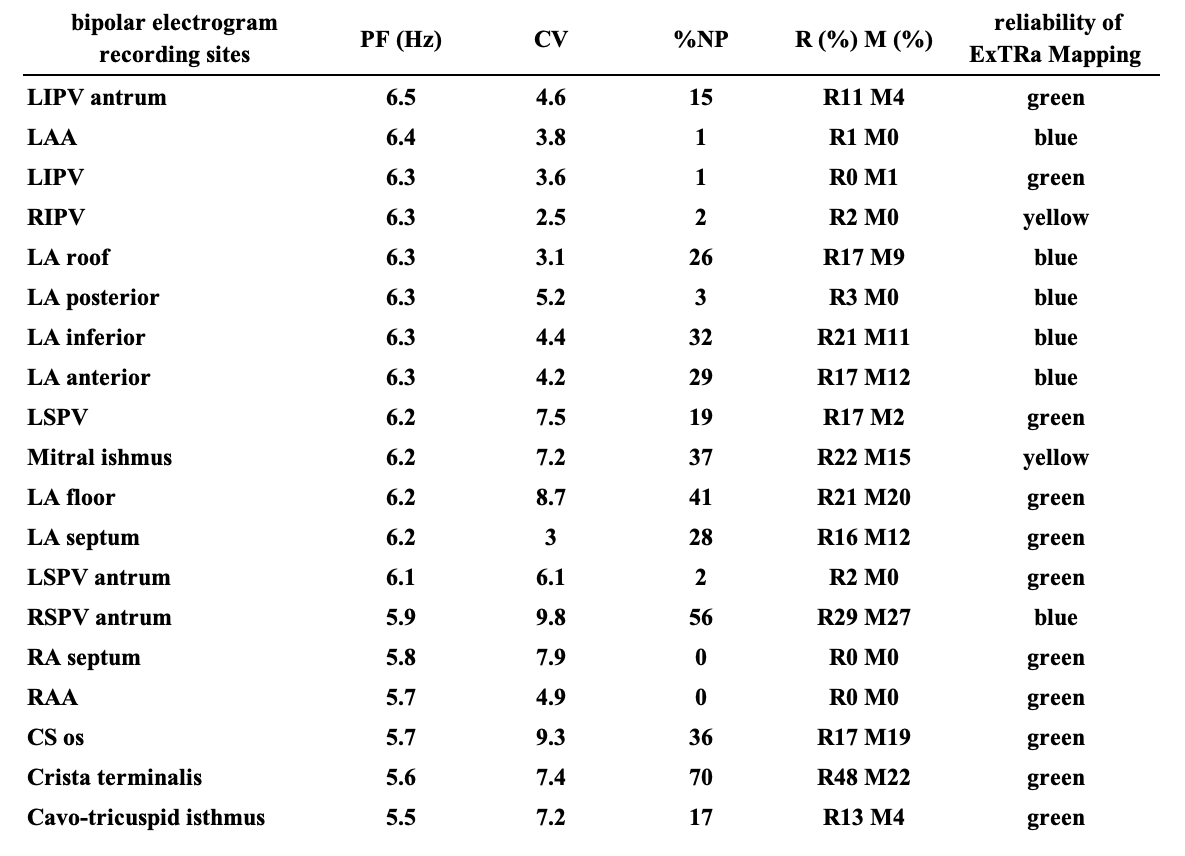


**Supplementary Table. Sites of the stable pseudo-frequency (sPF) in both atria**

The sites where sPF [pseudo-frequency (PF) with coefficient of variation (CV) <10)]^1^ was detected in both atria are shown in descending order. The meaning of the colors for the reliability of ExTRa Mapping is the same as in Figure 1.

%NP = nonpassively activated ratio; R = rotors; M = multiple wavelets; LAA = left atrial appendage; RIPV = right inferior pulmonary vein; LA = left atrium; LSPV = left superior pulmonary vein; RSPV = right superior pulmonary vein; CS = coronary sinus.

**
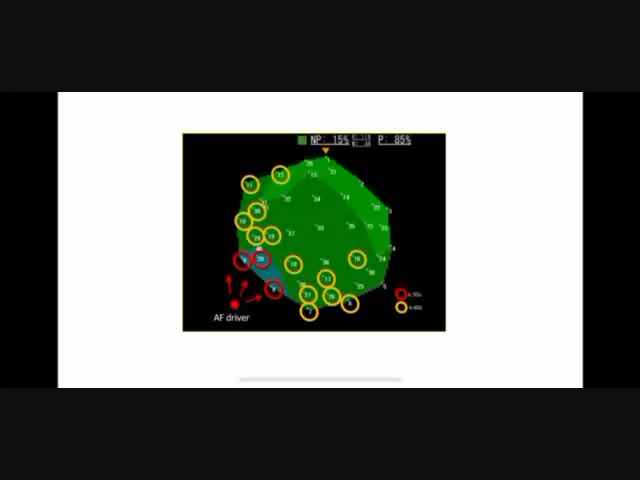
**

**Supplemental Movie 1**

Movie of representative atrial fibrillation (AF) wave dynamics at left inferior pulmonary vein antrum before pulmonary vein isolation. The movie shows the AF dynamics shown in Figure. 1. The indications of the numbers, white lines, open circles, red stars, and red arrows on the movie screen are the same as in Figures 1 and 2. The meaning of the green-colored square at the top of the phase map movie screen is the same as in Figure 1. The wavefront traveled intermittently and repeatedly from northwest to southeast. Abbreviations are the same as in Figures 1 and 2.

(Note: This is a still image. The movie will be played on the attached PowerPoint file.)

**
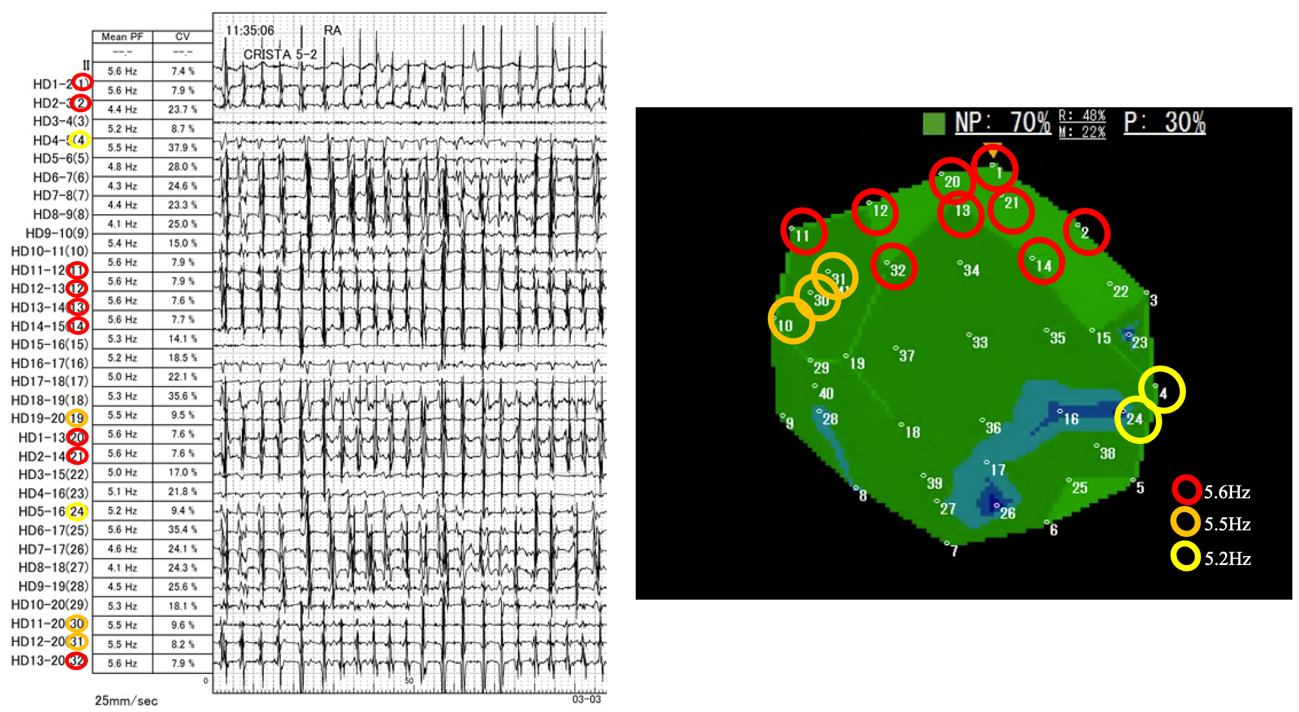
**

**Supplemental Movie 2**

Continuous wavelet transform analysis results and phase map movie screen at crista terminals during atrial fibrillation before pulmonary vein isolation, at which the highest nonpassively activated ratio of 70% was detected in both atria.

Left panel: Recordings from the top are electrocardiography lead II and physical bipolar electrograms recorded from the 20-pole spiral-shaped catheter (HD1-2 to HD13-20). The numbers in parentheses are the numbers 1 to 32 assigned to the physical bipolar electrograms. The second column shows the pseudo-frequency (PF), and the third column shows the coefficient of variation (CV). The sweep speed was 25 mm/s. Right panel: Phase map movie screen where the indications of the numbers on the screen are the same as those in Figure 1. The meaning of the green-colored square at the top of the phase map movie screen is the same as in Figure 1. The open circle represents stable PF (sPF= PF with CV <10)^1^ in both panels: red for 5.6 Hz, orange for 5.5 Hz, and yellow for 5.2 Hz. sPF at crista terminalis was far lower than the highest sPF of 6.5 Hz at left inferior pulmonary vein antrum.

Abbreviations are the same as those in Figures 1 and 2.

(Note: This is a still image. The movie will be played on the attached PowerPoint file.)
